# The association between single-nucleotide polymorphisms within type 1 interferon pathway genes and human immunodeficiency virus type 1 viral load in antiretroviral-naïve participants

**DOI:** 10.1186/s12981-024-00610-x

**Published:** 2024-05-03

**Authors:** Sara Bohnstedt Mørup, Preston Leung, Cavan Reilly, Brad T. Sherman, Weizhong Chang, Maja Milojevic, Ana Milinkovic, Angelike Liappis, Line Borgwardt, Kathy Petoumenos, Roger Paredes, Shweta S. Mistry, Cameron R. MacPherson, Jens Lundgren, Marie Helleberg, Joanne Reekie, Daniel D. Murray, for the INSIGHT FIRST and START study groups

**Affiliations:** 1 Centre of Excellence for Health, Immunity, and Infections, Copenhagen University Hospital, Rigshospitalet, Copenhagen, Denmark; 2 Division of Biostatistics and Health Data Science, School of Public Health, University of Minnesota, Minneapolis, MN USA; 3 Laboratory of Human Retrovirology and Immunoinformatics, Frederick National Laboratory for Cancer Research, Frederick, MD USA; 4 Chelsea and Westminster Hospital NHS Foundation Trust, London, UK; 5 Washington DC Veterans Affairs Medical Center and The George Washington University School of Medicine and Health Sciences, Washington, DC USA; 6 Center for Genomic Medicine, Copenhagen University Hospital, Rigshospitalet, Copenhagen, Denmark; 7 Kirby Institute, University of New South Wales, Sydney, New South Wales Australia; 8 Department of Infectious Diseases and IrsiCaixa, Hospital Universitari Germans Trias i Pujol, Badalona, Spain; 9 Institut Roche, Boulogne-Billancourt, France; 10 Department of Infectious Diseases, Copenhagen University Hospital, Rigshospitalet, Copenhagen, Denmark

**Keywords:** HIV-1, Viral load, Pathway analysis, *SKAT-O*, Type 1 interferon, Host genetics

## Abstract

**Background:**

Human genetic contribution to HIV progression remains inadequately explained. The type 1 interferon (IFN) pathway is important for host control of HIV and variation in type 1 IFN genes may contribute to disease progression. This study assessed the impact of variations at the gene and pathway level of type 1 IFN on HIV-1 viral load (VL).

**Methods:**

Two cohorts of antiretroviral (ART) naïve participants living with HIV (PLWH) with either early (*START*) or advanced infection (*FIRST*) were analysed separately. Type 1 IFN genes (n = 17) and receptor subunits (*IFNAR1, IFNAR2*) were examined for both cumulated type 1 IFN pathway analysis and individual gene analysis. *SKAT-O* was applied to detect associations between the genotype and HIV-1 study entry viral load (log10 transformed) as a proxy for set point VL; P-values were corrected using Bonferroni (P < 0.0025).

**Results:**

The analyses among those with early infection included 2429 individuals from five continents. The median study entry HIV VL was 14,623 (IQR 3460–45100) copies/mL. Across 673 SNPs within 19 type 1 IFN genes, no significant association with study entry VL was detected. Conversely, examining individual genes in *START* showed a borderline significant association between *IFNW1*, and study entry VL (P = 0.0025). This significance remained after separate adjustments for age, CD4^+^ T-cell count, CD4^+^/CD8^+^ T-cell ratio and recent infection. When controlling for population structure using linear mixed effects models (LME), in addition to principal components used in the main model, this was no longer significant (p = 0.0244). In subgroup analyses stratified by geographical region, the association between *IFNW1* and study entry VL was only observed among African participants, although, the association was not significant when controlling for population structure using LME. Of the 17 SNPs within the *IFNW1* region*,* only rs79876898 (A > G) was associated with study entry VL (p = 0.0020, beta = 0.32; G associated with higher study entry VL than A) in single SNP association analyses. The findings were not reproduced in *FIRST* participants.

**Conclusion:**

Across 19 type 1 IFN genes, only *IFNW1* was associated with HIV-1 study entry VL in a cohort of ART-naïve individuals in early stages of their infection, however, this was no longer significant in sensitivity analyses that controlled for population structures using LME.

**Supplementary Information:**

The online version contains supplementary material available at 10.1186/s12981-024-00610-x.

## Background

Disease progression among antiretroviral treatment (ART) naïve people living with HIV (PLWH) differs substantially [1], with both viral and host genetic factors (and their interaction) known to play an important role [2–4]. Human leukocyte antigen (HLA)-type is the most established host genetic factor associated with HIV progression [1, 3]. Additionally, variation within genes encoding chemokines and chemokine receptors, including CCR5D32, are known to be important for HIV susceptibility, primarily for HIV viral load and disease progression [1, 5]. However, common genetic variation, including HLA and *CCR5* SNPs, together with participant demographic variables are cumulatively estimated to explain only ~ 25% of VL variability [3, 6, 7]. From investigations of heritability in HIV-1 disease progression, the contribution of host genetics´ on HIV-1 viral load variability is estimated to be ~ 30% [8], of which about 5% is from loci beyond the well characterized variants in the major histocompatibility complex and *CCR5* regions of the genome. As such, a gap of knowledge remains. Other host genetic factors affecting HIV progression have been theorised to consist of either a combination of many small effect size variants or involve more complex interactions of variants across a genetic pathway [1, 7, 9]. Thus, here we explore the use of other approaches to investigate the role of host genetics in HIV pathogenesis beyond the traditional genome wide association studies (GWAS) [1].

Pathway analysis can assess genetic variants that may act in a functionally dependent manner and discover complex associations with an impact on the variability in disease progression [10]. In contrast to the multiple comparisons made in GWAS´, where variants are analysed individually, in pathway analysis groups of variants are analysed simultaneously, which increases the power to detect associations [10]. As such, a pathway analysis can exploit the advantages of analysing all variants (e.g., SNPs) contained within a genetic pathway. Knowledge of genetic pathways allows one to select SNPs from the distinct genes within the pathway to investigate their collective association with the outcome of interest. For HIV-1 disease progression, one such potential pathway is the type 1 interferon (IFN) pathway.

Type 1 interferons are important cytokines in the host´s immune system that play a role in combating viral infections. In SARS-CoV-2 infection, host genetic variation of type 1 IFN genes have been shown to impact the course of the infection [11–13]. For HIV, the type 1 IFN pathway is of particular interest [14–16]. In HIV, these cytokines not only mediate an early response as one of the major parts of the innate immune system, they also induce the transition of the innate immune system to the adaptive immune system [17, 18]. Further, type 1 IFNs induce important viral restriction factors in the large number of IFN stimulated genes. As such, the type 1 interferon pathway is a portion of the interferon pathway.

To identify IFN-induced HIV restriction factors M. OhAinle et al. [9] performed a CRISPR gene neutralization of these genes in CD4^+^ T cells. They found the inhibition of HIV-1 replication by IFNs were caused by combined actions of only a few IFN stimulated genes. These are known as HIV restriction factors, e.g., MxB, TRIM5alpha, IFITM1 and Tetherin.

Prior GWAS’ in HIV-1 infection including both European and multi-ethnic populations have not identified SNP level associations with HIV viral load (HIV-VL; a proxy of disease progression) in type 1 IFN genes [7, 19]. However, it may be that multiple SNPs across the individual type 1 IFN genes or the entire pathway impact HIV replication.

The stable level of viral load, defined as set point VL (spVL), which appears during the asymptomatic phase after acute HIV-1 infection, is a common prognostic measure for HIV infection severity, which can predict both infectiousness and rate of progression to disease [2]. Although, the level of HIV RNA before initiation of ART has been shown to differ significantly between groups based on sex, risk, age at- as well as year of seroconversion, and presentation during acute infection [20]. Using data from the Strategic Timing of Antiretroviral Treatment (*START)* trial [21], this study included a gene and pathway level analysis of the type 1 IFN pathway to assess whether accumulated genetic variation across individual genes or the entire pathway affects HIV replication in individuals with early infection. We then used data from the Flexible Initial Retrovirus Suppressive Therapies (*FIRST*) cohort [22] to validate the findings from the *START* trial in individuals with more advanced infection.

## Methods

### Participant population

The early infection cohort are participants from the *START* (NCT00867048) [23] trial, which enrolled HIV-1 positive individuals between 2009 and 2013 across a global network of clinical sites. At study entry, individuals were ART-naïve, aged > 18 years, and with CD4^+^ T-cell count > 500 cells/mm^2^. Relevant clinical data and biological material for research (among those who consented to specimen collection) were collected at the participant recruiting site. The *FIRST* cohort [22] was used for validation and included participants with more advanced HIV-1 infection, as there was no CD4 count requirement. The *FIRST* trial enrolled HIV positive participants from the US between 1999 and 2002, who were ART-naïve at study entry. The analyses in both cohorts were restricted to those who gave consent for genetic analysis.

### Outcome in *START* and* FIRST*

Viral load at study entry was used as the primary outcome measure in both the START and FIRST cohorts [21, 24].

### Genes included and SNP selection

The seventeen type 1 IFN pathway genes and their two receptors (*IFNAR1, IFNAR2*) located on either chromosome 9 or 21 were selected through an in-house software using pathway information (Table 1), such as gene members from the type 1 IFN pathway sourced from the Reactome database [25, 26]. Gene loci information was retrieved from Ensembl database [27, 28] through the BioMart R package [29]. Any SNPs overlapping the genes in the type 1 IFN pathway as well as being within a window of 2000 nucleotides upstream and downstream from each gene were included.

**Table 1:** Type 1 IFN genes and SNP counts

Gene	Entrez ID	Chromosome	SNP Count	Non-imputed SNP count	Imputed SNP count	Proportion of imputed SNPs (%)
*IFNA1*	3439	9	26	3	23	88.46
*IFNA2*	3440	9	12	1	11	91.67
*IFNA4*	3441	9	25	0	25	100.00
*IFNA5*	3442	9	17	1	16	94.12
*IFNA6*	3443	9	14	3	11	78.57
*IFNA7*	3444	9	14	1	13	92.86
*IFNA8*	3445	9	24	1	23	95.83
*IFNA10*	3446	9	44	2	42	95.45
*IFNA13*	3447	9	24	1	23	95.83
*IFNA14*	3448	9	13	0	13	100.00
*IFNA16*	3449	9	48	3	45	93.75
*IFNA17*	3451	9	25	1	24	96.00
*IFNA21*	3452	9	29	2	27	93.10
*IFNB1*	3456	9	18	1	17	94.44
*IFNW1*	3467	9	17	1	16	94.12
*IFNK*	56,832	9	31	5	26	83.87
*IFNE*	338,376	9	22	3	19	86.3
*IFNAR1*	3454	21	142	3	139	97.89
*IFNAR2*	3455	21	128	16	112	87.50
**Total SNPs**			**673**	**48**	**625**	

Bolded text denotes sum of SNP counts for that column

*IFN* interferon, *SNP* single nucleotide polymorphism

### Study cohort genotype data

Genotypic data from the *START* and *FIRST* trials has been described previously [21, 22]. Briefly, human DNA from study participants´ blood samples were genotyped using a custom Affymetrix Axiom SNP-array (including 770,558 probes), which was enriched using immune dysfunction related markers. Genome Reference Consortium Human Build 37 (Hg19) in Ensembl gene database [27, 28] was used for gene annotation.

### Quality control (QC) of genetic data

Participants were excluded if they had any of the following: sex mismatch, autosome SNP call rate less than 96%, duplicates, cryptic relatedness estimated by pairwise identity-by-descent (IBD) (pi-hat at least 0.90).

SNP Quality Control: SNPs with any of the following were kept for the GWAS: (a) recommended by Axiom Analysis Suite (Thermo Fisher Scientific), (b) >  = 90% reproducibility from the internal control Ref103, (c) For multiallelic sites, the SNP record with the higher quality score was kept and the remaining SNPs occurring at the same position were removed.

### SNP Imputation and post imputation QC

SNP imputation to the 1000 Genomes phase 3 [30] (genome build: GRCh37) was performed on raw genotyping data with the genipe pipeline [31] using PLINK (v2.00a3LM) [32] and SHAPEIT (version 2.5) [33] for phasing and IMPUTE2 (version 2.3.2) [34] for imputation. Imputed SNPs with a confidence score INFO <  = 0.8 and duplicates were removed. The threshold for minor allele frequency (MAF) was decreased from a limit of MAF > 5% in the previous GWAS [21] to MAF > 1% for the inclusion of rare SNPs. Remaining post-imputation QC was performed following the previous publication [21]. Briefly, SNPs fulfilling any of the following were excluded: (a) genotype missing rate > 10%, or (b) Hardy–Weinberg equilibrium p-value < 1 × 10^−6^. After SNP imputation, subjects fulfilling any of the following: (a) > 10% missing SNPs or (b) outside of expected heterozygosity (i.e., those with F values outside of 3 standard deviations above or below the mean) were also excluded.

### Calculation of PCAs and ancestry estimation

EIGENSTRAT [35] was used for principal components analysis (PCA) and the top 4 eigenvectors were included as covariates in the analysis to control for population stratification (Additional file 1). PCA was performed independently in each cohort based on SNPs passing QC and having MAF > 0.05 in line with the previous GWAS studies [21, 24]. For the START cohort only, continental-level ancestry was estimated using ADMIXTURE (https://dalexander.github.io/admixture/publications.html) and included 1000 Genomes samples (phase 3; n = 2504) as a reference. Inclusion criteria required that the SNPs were common to both the HIV cohort and 1000 genomes reference samples, array genotyped, non-ambiguous, biallelic, autosomal, and not present in high LD regions (as defined in https://github.com/cran/plinkQC/blob/master/inst/extdata/high-LD-regions-hg19-GRCh37.txt) or in HLA region chr6: 28477797–33448354 (hg19/GRCh37). PLINK (v1.9 and v2.0) was used for SNP and sample filtering (–maf 0.01, –geno 0.1, –hwe 1e−6, –mind 0.1) and pairwise-pruning (window size of 50 variants, step size of 5 variants, and r2 threshold of 0.2), leaving 159 406 SNPs for ancestry estimation. ADMIXTURE was run with K ancestral populations set to five, for consistency with the number of 1000 Genomes superpopulations.

### Statistical analyses

#### Preparation and performing SKAT-O analysis

The sequence kernel association test (SKAT) is a powerful method for gene level analysis of SNP array data and pathway analysis [10, 36]. *SKAT-O* [37–40] is an optimised variant, and is a bidirectional approach, which combines features of dispersion tests (i.e., allow for different directions of effect of rare variants on the outcome) and burden tests (i.e., assume effects of all rare variants having the same direction on outcome). Detection of associations through *SKAT-O* was between the genotypes collapsed into gene regions (here SNPs within type 1 IFN genes or receptors) or cumulatively in the pathway analysis of all 19 genes, and the phenotype; HIV-1 study entry VL.

To make use of this method, R scripts were implemented around *SKAT*-O where data formatting was performed to optimise the input data prior to using *SKAT-O* to test for associations with HIV-1 study entry VL. Briefly, START and FIRST cohort data were reformatted into three data sources; (1) Affymetrix SNP array probe identities remapped to Reference SNP cluster IDs (rsID) using chromosome and position (for imputed SNPs that did not have Affymetrix array probe id, their original rsID was used instead); (2) Participants´ allele counts for each SNP id; (3) Participants´ phenotype information and clinical data. These datasets were used to generate SNP sets lying within gene co-ordinates given by entrez ID [41] and the additional window size. Since Affymetrix array data used probe ID to identify SNP calls, data source (1) allowed the retrieval of Affymetrix probe IDs using rsID together with chromosome and position information. Data source (2) then allowed the access to retrieve participants´ genotype and subsequently, testing the cohort data against the phenotype of interest with corresponding covariates using data source (3). The scripts were written with R software (V. 4.2.0) in addition to using R SKAT-O package [42, 43] and named as GeneAnalysis_SKATO.R with GeneAnalysis_SKATO_Helper.R and checkDependencies.R as supporting scripts. Instructions for obtaining the docker image as well as access to the scripts are available at 10.5281/zenodo.8397641.

Entrez ID [41] for type 1 IFNs and *IFNAR1, IFNAR2* were provided as input data to the R scripts together with the upstream and downstream window size; covariates (gender and the first four principal component vectors), and the outcome (participants´ study entry HIV-1 viral load, log10 transformed); herein referred to as the main model. To test multiple genes (i.e., a gene set), Entrez IDs were grouped to be treated as one test set. *SKAT-O* runs were configured using the recommended default settings.

#### SNPs data and selection of SNPs included for SKAT-O analysis

Restrictions were placed to only include type 1 IFN genes and interferon-α receptors (*IFNAR1, IFNAR2*) in an initial investigation of type 1 IFNs. When SNPs were cross checked with gene loci in Ensembl [27, 28], a total of 673 SNPs were retrieved for the type 1 IFN genes, of which 625 (92.8%) were imputed SNPs (Table 1). Each SNP overlapped one of the 19 type 1 IFN pathway genes within an upstream/downstream window of 2000 base pairs of each gene’s loci (Table 2). The distribution of all 673 SNPs in IFN Genes are in Additional file 2.

**Table 2:** Demographics of participants at study entry

	Number of *START* participants. n (%)	Number of *FIRST* participants. n (%)
Total participants	2429	541
Age. median (IQR). years	37 (29–45)	38 (32–44)
Female sex	489 (20)	110 (20.3)
Race		
White	1398 (57.6)	142 (26.2)
Black	572 (23.5)	309 (57.1)
Hispanic	407 (16.8)	74 (13.7)
Other	52 (2.1)	16 (3.0)
Region of enrolment		
Europe and Israel	1135 (46.7)	0
United States	451 (18.6)	541 (100)
Latin America	413 (17.0)	0
Africa	339 (14.0)	0
Australia	91 (3.7)	0
Mode of HIV infection		
Injection drug use	45 (1.9)	65 (12.0)
Sex with same sex	1549 (63.8)	247 (45.7)
Sex with opposite sex	721 (29.7)	222 (41.0)
Sex with opposite sex (male proportion)	277 (38.4)	222 (100)
Sex with opposite sex (female proportion)	444 (61.6)	0
Other	114 (4.7)	7 (1.3)
CD4^+^ cell count		
Median (IQR). cells/µL	651 (585–760)	181 (43–344)
HIV RNA load		
Median (IQR). copies/mL	14,623 (3460–45100)	126,298 (34,332–343,784)
< 1000	287 (11.8)	8 (1.5)
1000–100000	1886 (77.7)	238 (44.0)
> 100,000	256 (10.5)	295 (55.5)
Hepatitis co-infection^a^		
Hepatitis B	54 (2.3)	29 (5.4)
Hepatitis C	107 (4.5)	92 (17.0)
Temporal information		
Time since diagnosis of HIV. median (IQL). years	1.1 (0.4–3.0)	N/A
Recent infection within 6 months^b^	164 (6.8)	N/A

*FIRST* flexible initial retrovirus suppressive therapies, *IQR* Inter quartile range. *N/A* not applicable, *START* Strategic Timing of Antiretroviral Treatment, QC: quality control

^a^For *START* Hepatitis B status was registered in 2341 participants and Hepatitis C status was registered in 2373 participants. In *FIRST* Hepatitis B status was registered in 518 participants, Hepatitis C status ass registered in all

^b^For *START* recent HIV infection was calculated based on participants´ self-reporting and a multi-assay algorithm used on baseline samples to verify these participants as having a recent infection (i.e. within 6 months before enrolment)

#### REGENIE method

SNP filtering, LD pruning, and REGENIE Step 1 were performed based on the full cohort (n = 2429). Step 1 of REGENIE [44] was run on biallelic, directly genotyped autosomal SNPs that were filtered with PLINK (–maf 0.01, –geno 0.1, –mind 0.1, –hwe 1e−15) and pruned for LD (window size of 1000 variants, step size of 100 variants, and r2 threshold of 0.9). Gender and the first 4 PCs (standardized to mean = 0 and standard deviation = 1) were included as covariates and the phenotype was log10 transformed. This step used a block size of 1000. SKATO analysis (Step 2) was run separately for the full cohort and two African groups that were defined based on geography (n = 339) or genetics (n = 525). The SKATO analysis used the same covariates and phenotype as in Step 1 and focused on 673 SNP variants in 19 genes of interest.

#### Sensitivity analysis

To assess the robustness of associations, sensitivity analyses were performed with additional covariates potentially related to viral load. These consisted of age; baseline CD4^+^ T-cell count and CD4^+^ / CD8^+^ T-cell ratio, and whether participants had a recent HIV infection (i.e., within six months before enrolment) were included separately in *SKAT-O* analysis. Recent HIV infection was calculated based on participants´ self-reporting and a multi-assay algorithm used on baseline samples to verify these participants as having a recent infection [45]. Further, since the START cohort is geographically diverse, we performed an additional sensitivity analysis using the REGENIE method, which used LME to control for population structures. Here, the subgroup analysis was based on participants´ geography (Europe, U.S., Latin America, Africa) and by gender (Table 2). Australia was not included due to low number of participants (n = 91). We also conducted a subgroup analysis of persons of African descent as defined by their ADMIXTURE fraction cut off (at least 0.7). Finally, we performed additional subgroup sensitivity analyses using the REGENIE method on the African subgroup defined by continent of recruitment and by ADMIXTURE fraction cutoff of at least 0.7.

#### Performing individual SNP association

Individual SNP level association was performed for any genes found to be significantly associated with HIV-1 viral load, using a linear model through PLINK (v2.00a3LM). When performing individual SNP association analysis, the same input parameters used in *SKAT-O* analysis with the addition of age were provided as covariates. Variance was standardized for age as a covariate parameter.

#### Multiple testing correction

Bonferroni correction was used to limit the family-wise error rate. A total of 20 *SKAT-O* analyses were performed: 19 individual analyses of the sets of SNPs within the type 1 IFN and receptor genes and one cumulative analysis including all type 1 IFN pathway SNPs (gene set). Using Bonferroni’s correction, the adjusted significance level was P < 0.0025 in correspondence to a total of 20 comparisons.

#### Validation

Using the *FIRST* cohort for validation, *SKAT-O* was applied for the type 1 IFN pathway analysis or in any type 1 IFN gene showing a significant p-value in *START* after adjustment for covariates.

## Results

### *START* participants

A total of 2440 *START* participants were eligible for inclusion in the analysis. However, since post imputation QC identified 11 participants with a heterozygosity coefficient (F value) outside ± 3 standard deviations from the mean, the total number of START participants included in this analysis decreased to 2429 (Table 2). Most participants were white (n = 1398, 58%), male (n = 1940, 80%), and enrolled in Europe (47%). The median age at study entry was 37 years (IQR 25 to 45). The median time since HIV diagnosis was 1.1 years (IQR 0.4–3.0) with a median viral load of 14,623 copies/mL (IQR 3460–45100) at enrolment. As per the enrolment criteria, all individuals had a CD4^+^ cell count > 500 cells/µL (median 651, IQR 585–760). The prevalence of hepatitis B and hepatitis C co-infections was low, with, respectively, only 2.3% and 4.5% of *START* participants being HIV-1/hepatitis co-infected.

### SNPs within *IFNW1 *region were significantly associated with study entry VL in *START* cohort

Cumulated *SKAT-O* analysis of type 1 IFN gene and receptor SNPs (i.e., all 19 genes treated as one set) showed no significant association (p = 0.15) with study entry VL (Table 3). In the individual gene analysis, *SKAT-O* detected a borderline significant association between higher levels of study entry VL and *IFNW1* (p = 0.0025). No other type 1 IFN gene was found to be significantly associated with study entry VL. Results were consistent in sensitivity analysis where additional covariates, including age, CD4^+^ T-cell count, CD4^+^/CD8^+^ T-cell ratio and recent infection were included (Table 3). The sensitivity analysis using the REGENIE method as an additional control for population structure were largely consistent with the main results, however the p-value for *IFNW1* was no longer significant after adjustment for multiple testing (p = 0.0244).

**Table 3:** SKAT-O P-values for type 1 IFN gene associations with the START cohort study entry VL

Gene	P-value ^a^	P-value adjusted for Age	P-value adjusted for CD4^+^ T-cell count	P-value adjusted for CD4 + /CD8 + T-cell Ratio	P-value adjusted for recent infection ^b^	P-value adjusted using PCs and linear mixed effect model
*IFNA1*	1.0000	1.0000	1.0000	0.8253	1.0000	0.9382
*IFNA2*	0.7521	0.7344	0.7521	0.6808	0.7758	0.3646
*IFNA4*	0.4216	0.4127	0.4216	0.3446	0.3359	0.8590
*IFNA5*	0.2959	0.2882	0.2959	0.2084	0.2644	0.9128
*IFNA6*	0.1250	0.1199	0.1250	0.1092	0.1074	0.7699
*IFNA7*	0.0548	0.0526	0.0548	0.0509	0.0602	0.1067
*IFNA8*	0.5932	0.5836	0.5932	0.5503	0.5800	0.6820
*IFNA10*	0.0496	0.0476	0.0496	0.0466	0.0371	0.5337
*IFNA13*	0.5685	0.5539	0.5685	0.4540	0.5004	0.5245
*IFNA14*	0.4506	0.4449	0.4506	0.5143	0.3705	0.7637
*IFNA16*	0.2339	0.2279	0.2339	0.1927	0.1851	0.6350
*IFNA17*	0.0203	0.0191	0.0203	0.0224	0.0200	0.2579
*IFNA21*	0.2072	0.1994	0.2072	0.1944	0.2070	0.6799
*IFNB1*	0.1767	0.1730	0.1767	0.1395	0.1896	0.4761
*IFNE*	0.3833	0.3647	0.3833	0.6926	0.3817	0.5965
*IFNK*	0.5016	0.5029	0.5016	0.7492	0.4640	0.6621
*IFNW1*	0.0025	0.0024	0.0025	0.0029	0.0025	0.0244
*IFNAR1*	0.5933	0.5900	0.5933	0.6599	0.5149	0.7593
*IFNAR2*	0.1503	0.1445	0.1503	0.0696	0.1497	0.2988
*All *^***c***^	0.1545	0.1473	0.1545	0.1115	0.1315	-

*IFN* interferon, *START* strategic timing of antiretroviral treatment, *VL* viral load

^a^The adjusted significance level was p < 0.0025 in correspondence to a total of 20 comparisons using Bonferroni

^b^Recent HIV infection was calculated based on participants´ self-reporting and a multi-assay algorithm used on baseline samples to verify these participants as having a recent infection (i.e., within 6 months before enrolment)

^c^All 19 genes were analysed as one

Two subgroup analyses were performed; one stratified by the four main geographical locations: Europe (N = 1135); Africa (N = 339); Latin America (N = 413); and the U.S. (N = 451), and the other by gender (Table 4). In subgroup analysis by geography, the association between *IFNW1* and HIV-1 study entry VL was only replicated in the African subgroup (p = 0.002). No significant association was detected between *IFNW1* and study entry VL in the other geographical subgroups. In the sensitivity analyses of the African subgroup, where we controlled for population structure using linear mixed effects models in addition to the PCs, the p-value for the association with VL increased to p = 0.0614. In the subgroup where persons of African descent were identified via Admixture, the p-value for the association with VL was slightly lower (p = 0.0348). In the analysis by gender, no significant outcome p-value was detected. However, it is worth noting 47% (N = 232) of the females included in the *START* study were enrolled in Africa compared with only 5% (N = 107) of the males.

**Table 4:** Subgroup analyses of *IFNW1* association with study entry VL using SKAT-O

Cohort	Subgroup Analysis	Group	No. of Participants	P-Value
*START*	Geographical	Europe	1135	0.325
		United States	451	0.181
		Latin America	413	0.683
		Africa	339	0.002
	Gender	Male	1940	0.064
		Female	489	0.021

*START* strategic timing of antiretroviral treatment, *VL* viral load

### Investigation and assessment of *IFNW1 *SNPs in *START*

To further investigate the SNPs within the *IFNW1* region, individual SNPs were extracted from imputation data. Seventeen SNPs were retrieved (Table 1). MAFs of the 17 *IFNW1* SNPs are shown in Table 5. One SNP, rs10964859, was in the three prime untranslated region (3´-UTR) of *IFNW1* gene. The remaining 16 SNPs overlapped either 2000 base pairs upstream or downstream from the *IFNW1* gene. *IFNW1* is in chromosome 9, located at positions 21,140,631 to 21,141,831 in the GRCh37 genome build.

**Table 5:** Alternate allele frequencies of the 17 *IFNW1* SNPs in *START* and GnomAD in percentages

rsID	Reference Allele	Alternate Allele	All ^a^	EUR ^a^	AFR ^a^	All^b^	AFR/AFR-AM ^b^	EUR^b^
rs10811479	A	T	14.94	16.96	14.16	14.42		
rs12005185	G	A	17.19	16.04	27.88	18.02		
rs7853363	A	G	34.97	34.58	47.05	64.03 ^f^		
rs28368130	A	G	10.78	12.25	5.75	13.84		
rs200450911	G	GA^c^	2.43	51.63	5.90	2.48		
rs79876898^d^	A	G	51.42	50.26	54.72	1.40	4.95	0.02
rs10964859	C	G	29.74	34.27	25.66	31.99		
rs77312138^d^	G	A	51.19	50.26	53.24	1.41	4.95	0.03
rs10757189	G	A	26.57	25.55	21.53	25.23		
rs10511694	C	T	28.19	26.08	26.99	27.67		
rs10964860	T	C	26.49	25.51	20.94	25.41		
rs28751285	G	A	18.86	31.17	12.09	19.77		
rs28751284	G	C	13.34	10.53	16.96	10.93		
rs199615728	G	A	34.77	34.71	37.76			
rs10964861	T	G	26.86	25.29	24.63	25.25		
rs1895673^e^	A	G.T.C	19.1	23.52	9.29	21.00		
rs141103108	GATT	G	1.69	50.53	4.87	4.14 ^e^		

*GnomAD* genome aggregation database, *SNP* single nucleotide polymorphism, *START* strategic timing of antiretroviral treatment, *EUR* European, *AFR* African, *AFR-AM* African American

^a^*START* participants. All (N = 2429), EUR (N = 1135) and AFR (N = 1940)

^b^GnomAD SNP frequency data. For Europeans allele frequencies, Finnish data was not included.

^c^One base insertion

^d^SNP MAF differ from total allele frequency due to composition of START population; rare in European and common in African populations

^e^Frequency for A-G substitution, A-T substitution is rare at 0.0002273% and A-C substitution is unavailable in GnomAD

^f^Total allele frequency > 0.5 and MAF is calculated as 1—TAF

### Linkage disequilibrium (LD) of *IFNW1 *SNPs in *START*

**Fig. 1:**
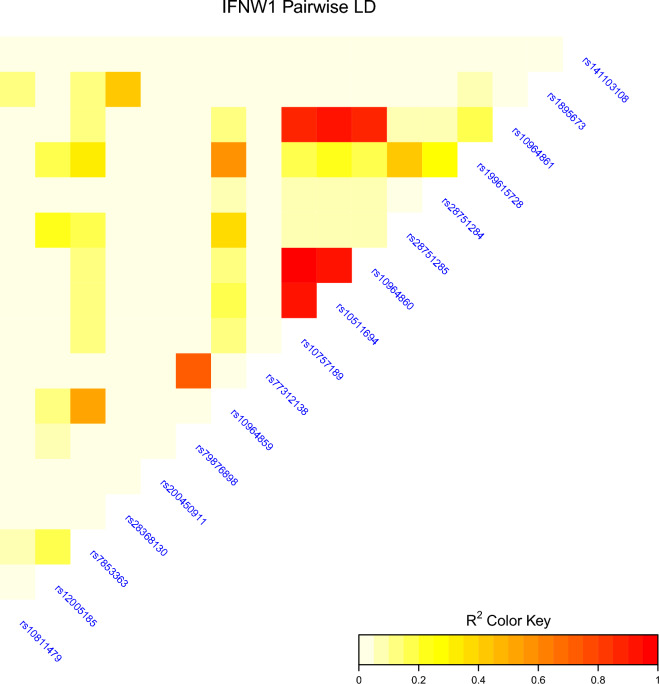
Heatmap illustrating *IFNW1* SNPs linkage disequilibrium (LD) in the *START* cohort. Legend indicates the strength with red representing high LD

We investigated Linkage Disequilibrium (LD) of the 17 *IFNW1* SNPs in the START cohort. Heatmap shows most of the SNPs were independent with a cluster of SNPs (rs10964861, rs10964860, rs10611694 and rs10757189) in LD (Fig. 1, Additional file 3).

Individual SNP association assessment with study entry VL in the *START* cohort by use of the same covariates (PC1, PC2, PC3, PC4 and gender) revealed no significant association among any of SNPs analysed, except for rs79876898, an A to G substitution (beta = 0.32, p = 0.002), which was found to be associated with higher viral load (Table 6). One of 17 SNPs, rs1895673 was included in the previously reported GWAS [21], while the remaining 16 SNPs were imputed and hence only examined here. The outcome of rs1895673 in the GWAS (p = 0.048, beta = − 0.059) was similar to the result for the SNP (p = 0.037, beta = − 0.062) in this study.

**Table 6:** Association analysis of 17 *IFNW1* SNPs with study entry VL in the *START* cohort

rsID	Reference allele	Alternate allele	Beta	Std. Err	T-statistic	P-value^a^
rs79876898	A	G	0.318	0.103	3.090	0.002
rs7853363	A	G	0.069	0.025	2.790	0.005
rs77312138	G	A	0.277	0.111	2.481	0.013
rs1895673	A	G.T.C	− 0.062	0.030	− 2.081	0.037
rs200450911	G	GA^b^	− 0.153	0.077	− 1.982	0.048
rs12005185	G	A	0.046	0.032	1.465	0.143
rs10811479	A	T	− 0.034	0.033	− 1.028	0.304
rs28368130	A	G	− 0.037	0.037	− 1.001	0.317
rs141103108	GATT	G	− 0.078	0.092	− 0.840	0.401
rs10964861	T	G	− 0.017	0.026	− 0.637	0.524
rs10964859	C	G	0.016	0.026	0.611	0.541
rs28751284	G	C	− 0.018	0.035	− 0.514	0.607
rs28751285	G	A	0.013	0.030	0.433	0.665
rs10757189	G	A	− 0.011	0.027	− 0.426	0.670
rs10964860	T	C	− 0.010	0.027	− 0.390	0.697
rs10511694	C	T	− 0.007	0.026	− 0.269	0.788
rs199615728	G	A	− 0.003	0.024	− 0.125	0.901

*Std. Err.* standard error, *SNP* single nucleotide polymorphism, *START* strategic timing of antiretroviral treatment, *VL* viral load

^a^Results ordered by p-value

^b^One base insertion

### Validation in *FIRST* cohort

For the *FIRST* cohort, 544 participants had available genotyping data. Three participants were excluded due to an F value outside ± 3 standard deviations from the mean. This resulted in a final cohort of 541 participants. Similar to the *START* cohort, the majority of the participants in the *FIRST* cohort were male (N = 431, 80%) and the median age was 38 years old (IQR 32–44 years old). In contrast, self-reported ethnicities of this cohort were primarily black (N = 309, 57%) with the next largest group being white (N = 142, 26%). All participants were enrolled from the US. Time since HIV diagnosis was unavailable. The *FIRST* trial consisted of ART naïve individuals with advanced disease, and this is reflected in study entry characteristics, particularly median HIV VL, which was higher in the *FIRST* cohort when compared to the *START* cohort (126,298 vs 14,623 copies/mL). The prevalence of hepatitis B (5%) and hepatitis C co-infections (17%) at study entry in *FIRST* was also higher in comparison to *START* (2% and 5%, respectively).

No significant association was detected between *IFNW1* and study entry VL (p = 0.1665) in *FIRST* participants when tested under the same conditions as *START* participants (Table 7). Also, sensitivity analysis after adjustment for age and CD4^+^ T-cell count at baseline, respectively, did not lead to a significant outcome of *IFNW1 SKAT-O*. As we did not observe associations using the main model, we did not conduct subgroup analysis of the *FIRST* participants. Likewise, we did not investigate associations of single *IFNW1* SNPs with HIV VL in the *FIRST* cohort.

**Table 7:** SKAT-O p-values of *IFNW1* association with the study entry VL in the *FIRST* cohort

Gene	P-value	P-value adjusted for Age^a^	P-value adjusted for CD4^+^ T-cell Count
*IFNW1*	0.1665	0.1910	0.3201

*FIRST* flexible initial retrovirus suppressive therapies, *VL* viral load

^a^Additional covariates potentially related to viral load were included separately one in *SKAT-O* sensitivity analysis; age and baseline CD4^+^ T-cell count

## Discussion

In this study, we conducted a pathway analysis with *SKAT-O* using phenotype and genotype data from a diverse cohort of ART naïve PLWH to access associations between type 1 IFN pathway genotypes and HIV-1 study entry VL. While the pathway analysis did not show a significant association in analysis of individual type 1 IFN genes, a borderline significant association between *IFNW1* and study entry VL (p = 0.0025) was detected that was stable in sensitivity analysis when additional covariates potentially related to HIV-VL were added. However, in the sensitivity analysis utilising linear mixed effects models to control for population structure, the p-value increased above the multiple testing corrected threshold in the full START cohort. Subgroup analysis indicated this association could be driven by participants from Africa, although again, the signal was lower in subgroup analyses using linear mixed effects models to control for population structure. Therefore, this signal warrants further investigation in a cohort from this region. In analysis of *IFNW1* single SNP associations, only rs79876898 was significantly associated with study entry VL (higher if G than A).

This study builds on a previous GWAS in *START* [21] by incorporating a gene and pathway level analysis of the type 1 IFN pathway. No significant association with HIV study entry VL was observed in analysis of the type 1 IFN pathway. However, the association with study entry VL of one type 1 IFN gene, *IFNW1,* which encodes the interferon omega-1 protein, indicates that the combination of the 17 *IFNW1* SNPs may influence HIV VL. Thus, genetic variation in *IFNW1* may be of importance for downstream type 1 IFN signalling and thereby impact HIV-1 replication. *SKAT-O* assesses bidirectional associations, but the methodology does not provide any information on the direction of the potential effect of *IFNW1* on viral replication (upregulate/downregulate). To gain some knowledge on this we subsequently investigated the individual SNP level associations in the *IFNW1* gene. Here, one SNP, rs79876898 was found to be associated with higher viral load. This SNP was not examined in the previous GWAS [21], as that study did not impute SNPs. We additionally searched for this SNP in other GWAS publications [3, 46] that focused on the association of variants with HIV viral load. Chromosome 6 was a recurring finding in terms of association with HIV viral load. However, rs79876898 (located in chromosome 9) was not identified in any of the studies. Furthermore, inspection of dbSNP [47] databases returned records for rs79876898; but no publications were linked to the SNP. ClinVar [48], a database that records association of SNPs to clinically relevant diseases also did not yield any records. Hence, the identification of rs79876898 to be associated to higher viral load appears to be novel, but the lack of prior associations and the potential confounding by population structures mean that this association should be interpreted cautiously.

*IFNW1* is known to have antiviral, anti-proliferation, and antitumor effects [49]. In one in vitro study *IFNW1* was shown to be a potent inhibitor of HIV replication, and compared with *IFNA2*, HIV-1 protein synthesis was more sensitive to *IFNW1* in this study [50].

Three of the *IFNW1* SNPs had prior citations in PubMed [51]; rs10757189, rs10511694, and rs10964859 [52–55] but not related to HIV. rs10964859 C > G is an imputed SNP located in 3´-UTR of *IFNW1* and having MAFs of 35%, 26% and 19% in European, African and Latino/admixed American populations, respectively [56]. In studies investigating cancer patients, rs10964859 has been noted as a potential regulatory variant, which may impact gene expression due to loss of miRNA binding [52–54]. The two additional SNPs, rs10757189 [55], rs10511694 [53, 55] have previously been reported to be associated with outcomes related to cancer; melanoma (rs10511694) and colorectal cancer (rs10757189, rs10511694). We did not find any prior citations for the remaining SNPs. By investigation of LD in the *START* cohort, we found rs10757189 and rs10511694 were in LD (r^2^ = 0.913). rs79876898, which was significantly associated with study entry VL in *START,* was not in LD with any of the three *IFNW1* SNPs, that had been published. Future studies may lead to improved knowledge of whether these SNPs impact type 1 IFN gene expression or type 1 IFN proteins in vitro or in vivo.

In subgroup analysis the association of *IFNW1* with study entry VL appeared to be driven by participants of African descent. Different gender compositions between geographical subgroups may affect the results of *SKAT-O* subgroup analysis, since VL is known to be affected by gender [57]. Further, different HIV-1 subtypes between geographical subgroups may have affected the VL levels [58]. The finding related to a possible association between variation in *IFNW1* and study entry VL in *START* [23] was not validated in the *FIRST* cohort [22]. In addition to having a much lower sample size, the lack of consistency of findings between these cohort may be explained by the composition of the two cohorts. *FIRST* exclusively enrolled in the U.S, compared to *START* where only 20% were from the U.S. Additionally, individuals in *FIRST* were more advanced in their HIV infection than *START* participants (with substantially lower CD4^+^ T-cell count and higher HIV-VL at study entry). The sensitivity analysis adding baseline CD4^+^ T-cell count as a covariate did not alter the outcome of *IFNW1 SKAT-O* in the FIRST cohort. However, the stages of progression in HIV-1 infection seen in *START* [23] and *FIRST* [22] might be controlled by different type 1 IFN genes. Additionally, the proportions of participants coinfected with Hepatitis B or C differed between *START* and *FIRST* participants. Notably, the proportion of *FIRST* participants co-infected with HCV was 17%. However, in a review and meta-analysis, HIV/HCV co-infection was not found to lead to a significantly higher HIV VL [59].

The potential association of *IFNW1* with HIV-1 VL was also not detected in a study by McLaren and colleagues [7], which used *SKAT-O* to identify associations between all human genes and HIV progression. The cohort investigated by McLaren consisted of 962 participants, and thus included a similar sample size compared with the sample size of Europeans in our study. However, since this study was conducted in a European cohort, it would not have observed associations observed in individuals of African descent. Further, the majority of *IFNW1* SNPs (16/17) were imputed and located outside the coding region. These SNPs would therefore not be included in the exome sequencing used by McLaren et al. and cannot be directly compared with their study. Since the *SKAT-O* outcome of *IFNW1* was not validated in the *FIRST* study or elsewhere, it is important to emphasize the association of *IFNW1* with HIV-1 VL reported here is lacking validation and should be interpretated cautiously. Without validation, analysis of selected candidate genes or a candidate pathway is at risk of type 1 error [60]. Therefore, an investigation of whether the association of gene level variation in *IFNW1* with HIV VL is true shall preferably be in a cohort of participants similarly at an early stage of HIV-1 infection and including participants of African descent. A recent study did explore the genetic contribution to HIV-1 spVL in a cohort of PLWH from Africa (n = 2682) at the SNP level [61]. This study did not observe any SNP level associations outside the HLA region and the *CHD1L* gene. Although, this study did not assess gene level associations and used a more stringent p-value cut-off than used in our study. However, taken together, the lack of current validation at both the SNP and gene level mean that the associations observed in our study should be interpreted cautiously.

There were several limitations to this study that warrant discussion. A common limitation to genetic studies is the diversity and the frequencies of genotypes and small sample sizes across the ethnic subpopulations. In our study, and particularly in the demographically diverse START cohort, we are challenged by this. We attempted to control for potential demographic and environmental biases in three ways. Firstly, by including principal components in the main model. Second, by conducting subgroup analyses where participants are separated into more demographically homogenous groups. Thirdly, by performing sensitivity analyses that utilised linear mixed effects models to control for population stratification, in addition to principal components. These methods are suggested to be superior to the use of PCs alone to control for population structure in genetic association studies. In our results, we see that the p-value for the IFNW1 association with HIV-VL increase above the multiple testing threshold used in this study. This indicates that there may be biases introduced by population structures that account for some of the signal in the main model. Due to the limited statistical power considering fewer participants within subgroups, we did not investigate further participant subgroups based on country of enrolment. As a common challenge in genetic studies, the sample size of our cohort limits our ability to analyse rare variants, as well as providing sufficient statistical power to analyse diversity in ethnicity of study participants. Use of SNP array genotype data, as opposed to having e.g., genome wide sequencing data, which would provide further information on rare variants, is also a limitation. Further, our study did not assess type 1 IFN gene expression or serum type 1 IFN measures. Finally, it is important to note our study did not assess or incorporate knowledge of genetic variants in *CCR5* (including CCR5D32) and HLA genes known to affect viral load. Thus, the association detected of *IFNW1* SNPs and study entry VL may be due to variation in genes prior known to affect VL. A strength of the *START* study is in the cohort size of European participants.

To conclude, in cumulated *SKAT-O* analysis on imputed type 1 IFN SNPs from a cohort of ART naïve HIV-1 positive participants, we did not detect an association with HIV disease progression. However, for the cohort of HIV-1 positive individuals in early stages of their infection, we detected an association between *IFNW1,* and HIV-1 study entry VL. This association was most apparent in persons enrolled in Africa. The observed association was no longer significant after more stringent control for population structure and the results observed here should be interpreted cautiously without additional validation.

### Supplementary Information

**Additional file 1.** Pairwise principal component plots (PCs 1-5) illustrating population stratification for (A) START and (B) FIRST cohort. The START cohort are overlayed with ancestry estimations.

**Additional file 2.** Table of SNPs in type 1 IFN Genes (N=673).

**Additional file 3.** Linkage Disequilibrium Heatmap values for Fig. 1.

## Data Availability

Due to data protection regulations and patient confidentiality concerns, the datasets analyzed in this study are only available via reasonable request to the corresponding author and approval from the INSIGHT Scientific Steering Committee. Software used for this study was written in R and can be obtained at https://doi.org/10.5281/zenodo.8397641 under the conditions outlined in GNU GPL-3.0 license.

## Abbreviations

ART: Antiretroviral
*FIRST*: Flexible initial retrovirus suppressive therapies
GWAS: Genome wide association study
HIV-VL: HIV viral load
HLA: Human leukocyte antigen
IFN: Interferon
LD: Linkage disequilibrium
PCA: Principal components analysis
PLWH: People living with HIV
QC: Quality control
SpVL: Set point viral load
*SKAT-O*: Sequence kernel association test, optimised variant
SNP: Single nucleotide polymorphism
*START*: Strategic timing of antiretroviral treatment trial
VL: Viral load
