# Supplementary material for: The association between single-nucleotide polymorphisms within type 1 interferon pathway genes and human immunodeficiency virus type 1 viral load in antiretroviral-naïve participants
Source: AIDS Res Ther. 2024 May 3;21:27. doi: 10.1186/s12981-024-00610-x (PMC11067292; doi:10.1186/s12981-024-00610-x)
Supplement: Supplementary file 1 — Additional file 1. Pairwise principal component plots (PCs 1-5) illustrating population stratification for (A) START and (B) FIRST cohort. The START cohort are overlayed with ancestry estimations. [file 12981_2024_610_MOESM1_ESM.docx]

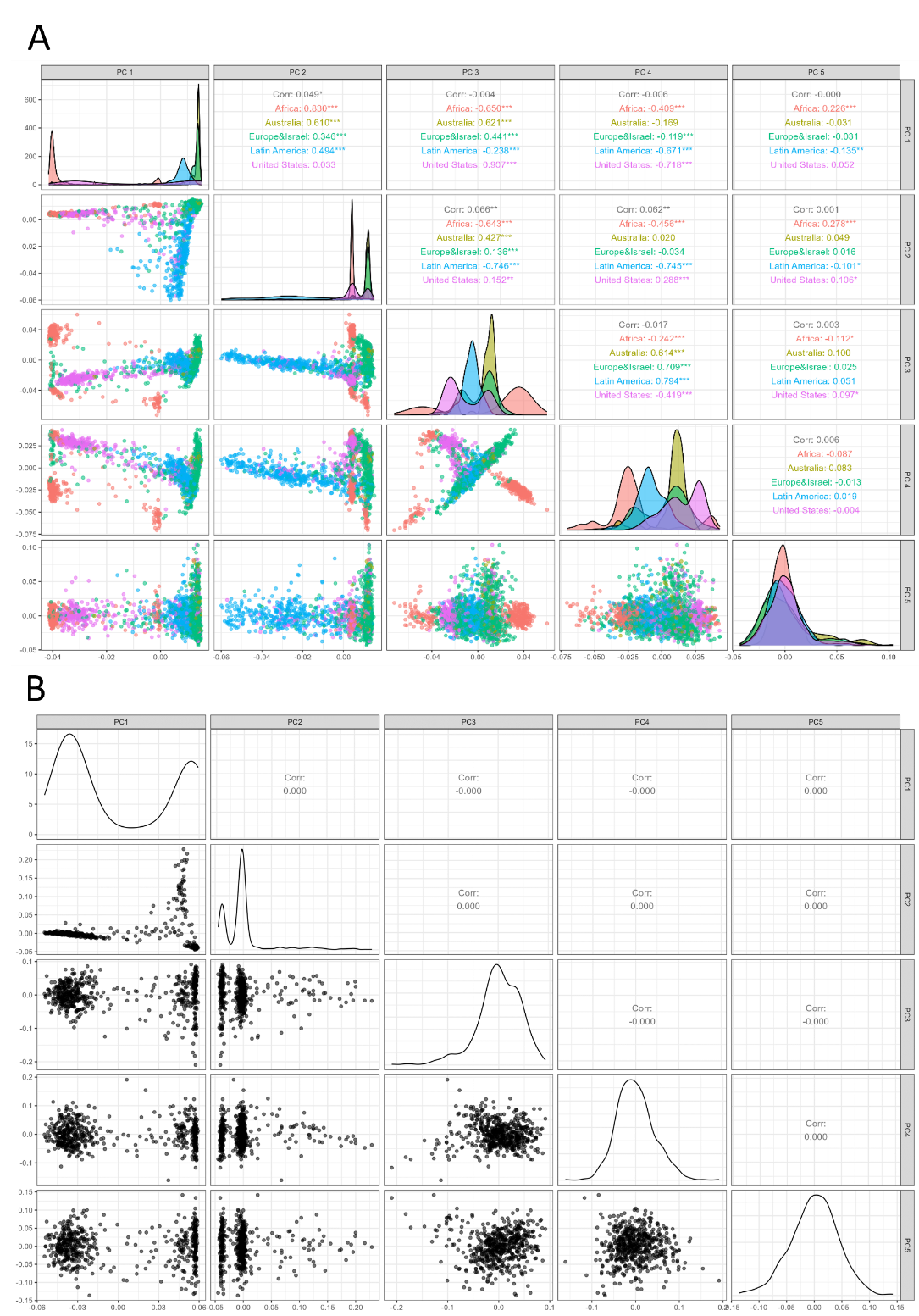


Additional file 1 - Pairwise principal component plots (PCs 1-5) illustrating population stratification for (A) START and (B) FIRST cohort. The START cohort are overlayed with ancestry estimations.
